# Supplementary material for: Are carbon emissions trading and green financial instruments synergistic? -Comprehensive quantitative research based on content analysis
Source: PLoS One. 2024 Mar 7;19(3):e0298601. doi: 10.1371/journal.pone.0298601 (PMC10919601; doi:10.1371/journal.pone.0298601)
Supplement: S1 Data — (ZIP) [file pone.0298601.s001.zip › Data/地级市Z_F工作报告.zip!/╣π╢1⁄2/╔╟═╖╩╨/└┤╫╘╣1⁄2╓┌║┼í╛┬φ┐╦╩2╛▌═°í┐_2006.docx]

各位代表：

我代表市人民政府向大会作政府工作报告，请予审议，并请政协各位委员和其他列席人员提出意见。“十五”时期回顾和2005年工作“十五”时期是我市在负重中拼搏、在困境中奋起、在继承中发展的五年。市政府在省委、省政府和市委的正确领导下，坚持以邓小平理论和“三个代表”重要思想为指导，认真贯彻落实科学发展观和建设社会主义和谐社会重要战略思想，立足当前，着眼长远，凝心聚力，真抓实干，着力解决关系经济社会发展的主要问题和矛盾，经济保持上升态势，社会事业取得新进展，精神文明和民主法制建设取得新成效，出现了稳定发展的新局面。

经济呈现较快恢复性增长。全市生产总值从2001年负增长1.9%后逐步上升，2002年增长5.7%，2003年增长8.8%，2004年增长11.1%，2005年增长11.3%，年均增长6.9%。五年间，地方财政一般预算收入年均增长9.2%，工业总产值年均增长7.8%，社会消费品零售总额年均增长9.5%，全社会固定资产投资额年均增长3.2%，外贸出口总额年均增长4.2%，金融机构各项存款余额年均增长12.7%。

产业结构逐步优化。一、二、三次产业增加值比重由“九五”期末的8.7 48.3 43.0发展为6.8 51.5 41.7。规模经济特征日益显现，形成纺织服装、化工塑料等八大支柱产业和31个工业产业集群，规模以上工业总产值占全部工业总产值的比重比“九五”期末提高16.5个百分点。拥有中国名牌产品8个、中国驰名商标5件、国家免检产品7个，拥有“中国玩具礼品城”、“中国针织内衣名镇”等7个区域性品牌称号，被授予“中国品牌经济城市”称号。农业产业化、标准化步伐加快。旅游、会展、物流、信息等现代服务业蓬勃发展。

城市发展新格局初步形成。2003年实施行政区划调整，市区面积从301平方公里扩大到1956平方公里。初步形成东西扩延、南北对称、中心带动、区域协调发展的城市格局，一批中心镇的规划建设得到加强。规划东部城市经济带、工业经济带和生态经济带，为城市发展和工业发展提供广阔空间。一批重点基础设施项目竣工投入使用，城市综合竞争力和辐射能力进一步提升。

人民生活质量有新改善。中心城区居民人均可支配收入年均增长4.9%。城乡居民储蓄存款余额年均增长15.9%。物价基本保持稳定。供电供水、道路交通、医疗卫生、教育文化和社会保障得到改善。荣获“国家环境保护模范城市”、“中国优秀旅游城市”、“全国双拥模范城”和“全国残疾人工作示范城市”称号。我市运动员在2004年雅典奥运会上荣获2枚金牌。2005年是“十五”计划的最后一年，我们围绕年初政府工作报告提出的“六个统一”、“四个突破”的要求，把努力做到“远”“近”结合、“大”“小”结合、“内”“外”结合、“快”“好”结合作为全面贯彻落实科学发展观的重要举措，积极进取，奋力拼搏，经济建设、政治建设、文化建设和社会建设取得新成绩，市十一届人大三次会议提出的主要目标任务顺利完成。———经济增长速度与质量、效益同步提升

紧紧扭住经济建设这个中心不放松，大力实施工业强市、科教兴市和品牌带动战略，实施投资拉动、出口拉动、消费拉动，加强经济运行监测调节，强化财政税收管理，国民经济实现较快增长。全市生产总值650.8亿元，增长11.3%；地方财政一般预算收入29.4亿元，增长19.4%。

工业经济加快发展。实现工业总产值1289.4亿元，增长14.2%。传统工业得到改造提升，电子信息和机械装备等行业发展势头良好，高新技术产品产值达193亿元。澄海区被省确定为创建区域国际品牌试点地区。第三产业增加值增长9.2%。交通运输稳步增长，港口货物吞吐量、集装箱吞吐量分别增长10.1%和29.1%。旅游业总收入增长14.1%，房地产业保持适度增长。

发展活力不断增强。外贸出口快速增长，全市进出口总额49.6亿美元，增长18.7%，其中出口总额31.8亿美元，增长25%。实际吸收外商直接投资金额1.1亿美元，增长35.7%。全社会固定资产投资额155.8亿元，增长18%。民间投资日趋活跃，占全社会固定资产投资额五成以上。首届潮商大会成功举办，大型经贸活动取得良好成效。消费市场持续繁荣，全市社会消费品零售总额344.8亿元，增长17%。

经济效益有所提高。全市地方财政一般预算收入中税收收入按可比口径增长14.8%，高于生产总值增幅。克服石油、原材料涨价等不利因素的影响，工业经济效益继续向好。全市规模以上工业实现利润总额27.4亿元，增长11.1%；工业经济效益综合指数142.3%，提高6.2个百分点。———农业农村经济稳步发展

农业产业化、标准化、现代化不断推进。完成农业总产值85.4亿元，增长2.7%。粮食播种面积108.5万亩，产量50.8万吨，完成粮食工作考评任务。全市农业渔业龙头企业带动17.7万户农民增加收入1.9亿元。2项农业科技成果获省部级奖励，引进推广46个农作物新品种。新修订26项农业渔业地方标准，新增10个农业渔业标准化示范区。白沙农业现代化示范区建设走在全省前列。农产品加工和流通水平不断提高，水产品出口创汇连续4年居全省首位。市农业科技园成为全国农业旅游示范点。

农田基础设施逐步改善。耕地保护不断加强。农业综合开发土地治理项目继续推进，一批中低产田得到改造。海门渔港被批准为国家中心渔港建设项目。

城镇化建设取得新进展。制定城镇化发展纲要。中心镇规划建设步伐加快，市政基础设施、公共服务设施不断完善，辐射带动作用增强。农村工业化进程加快，涌现一批专业镇、特色镇，转移农村富余劳动力11.9万人。

农村综合配套改革积极推进。免征农业税。清理废止涉农收费项目18项，降低涉农收费标准12项。农村会计代理制逐步铺开，财务管理得到加强。第二轮土地承包工作进展顺利。———城市规划建设有新进展

规划体系不断完善。城市发展概念性规划综合成果编制完成，城市与产业发展战略定位研究取得初步成果。开展新溪、外砂分区规划。市、县、镇三级土地利用总体规划调整修编工作全面展开。汕头港总体规划着手修编，广澳港区总体规划已经完成，干线公路网规划、公路运输枢纽规划、城市公交规划通过评审。海洋功能区划已上报省政府审批。

三条经济带规划有力推进。城市经济带已完成新津河口、外砂河口治理开发工程项目建议书编制和上报，开展东部城市经济带城市设计编制工作。工业经济带已完成控制性详细规划编制，在招商经贸活动中重点推介。生态经济带规划的调研工作着手进行。

一批能源交通市政设施建设进展顺利。500千伏汕头变电站1号主变扩建工程和华能电厂二期工程竣工投产，金鸿公路、深汕高速公路大修一期工程、汕头港航道整治二期工程等项目基本完成。广澳港区一期工程、金凤路桥、国道324线澄海路段3座大桥扩建等项目继续推进，广澳港区5万吨级航道和南澳大桥项目正式立项，揭海公路、省道谷饶－贵屿路段工程等项目开工建设。雷打石生活垃圾处理场二期工程应急填埋片区竣工投入使用，第二过海水管续建。各区县污水处理和垃圾处理等环保基础设施建设加快推进。

一批城乡防灾减灾水利工程建设顺利完成。完成水利建设投资2.5亿元，维修加固江海堤围80公里，除险加固小型水库25座。牛田洋海堤、秋风岭水库、外砂桥闸重建工程、乌桥岛堤围达标加固工程基本完成，棉北海堤、东里桥闸重建工程建设顺利推进。

城市面貌不断改善。城市绿地系统总体规划、城市照明规划、中心城区户外广告专项规划编制完成。韩江路全段贯通，杏花街“水浸街”改造、蓝水星主题公园周边道路建设、龙湖沟整治等项目进展顺利。城市环境综合整治力度加大，时代广场等重点公共场所管理得到加强。中心城区新增绿地面积11.4万平方米，人居环境继续改善。———改革迈出新步伐

民营经济发展壮大。贯彻落实国务院《关于鼓励支持和引导个体私营等非公有制经济发展的若干意见》。制定《汕头市中小企业信用担保机构扶持补贴暂行办法》。民营经济上规模上档次，全市规模以上民营工业企业843家，省级民营科技企业232家。民营工业实现产值789亿元，增长14.6%，占全部工业总产值的61.2%。

国有资产监管新体制初步建立。成立市国有资产监督管理委员会，建立市属国有企业改革工作联席会议制度，国企改革稳步推进。完成9家市属国有企业改制，安置职工2305人。澄海区和南澳县国有粮食企业改革基本完成。上市公司股权分置改革进展顺利。

投融资体制改革有所突破。出台鼓励投资城市基础设施和公用设施政策。市医疗特种废弃物处理中心建成投入使用，龙珠水质净化厂项目采用BOT、TOT运作成功。国家开发银行贷款第一批11个政府建设项目逐步推进，第二批10个项目全部动工建设。成立中心城区土地交易中心和土地储备中心，全面推行经营性用地招标、拍卖、挂牌出让制度，出让土地638亩，清理闲置土地2639亩。

各项改革进一步深化。调整国土资源、规划、房产管理和文化广电新闻出版管理体制，组建文化市场、国土资源综合行政执法队伍。推进公共财政体系建设，全市有422个行政事业单位纳入国库集中支付管理。农村信用社改革试点工作顺利开展，潮阳、澄海农信联社获得央行票据发行，南澳农信联社完成增资扩股任务。汕头市商业银行等地方金融机构资产保全清收力度加大，最终处置工作稳妥推进。水价、电价改革进一步深化，路桥收费年票制改革取得初步成效。———社会事业全面进步

科技综合实力增强。通过第二次全国科技进步考核，获省科技进步奖11项。金平区、澄海区被评为“全国科技进步先进区”。新增科技成果77项，推广转化科技成果112项。被确定为“国家知识产权试点城市”，新增专利授权2277件。以企业为主体的技术创新体系建设加快，新增国家和省级高新技术企业41家。组建国家级产业基地2个，新增省级技术创新试点专业镇6个、工程技术研究开发中心2个。技术市场不断完善。信息化建设进一步推进，涌现了一批国家和省级示范工程和单位。

教育和人才事业保持发展。“普九”成果得到巩固提高，高中办学规模扩大，职业教育、成人教育和民办教育稳步发展。中小学校舍建设和布局调整步伐加快，竣工交付使用项目66个，新增学位3万多个。优质基础教育规模扩大，新增省一级学校5所，市一级学校10所。汕头大学和汕头职业技术学院办学水平进一步提高。修订出台优秀人才引进政策，人才引进、培养、流动机制不断完善，人才市场工作进一步加强。

精神文明建设取得新成效。隆重举行清明节祭奠革命烈士、抗日战争胜利60周年系列纪念活动，以爱国主义为核心的民族精神和“海纳百川、自强不息”的汕头精神得到弘扬和实践。现代公民教育初显成效，全民读书活动月活动广泛开展。青年学生和未成年人思想道德建设得到加强，关心下一代工作力度加大。群众性精神文明创建活动稳步推进，全市有7个单位被评为全国精神文明建设先进单位。国防教育、国防后备力量建设和双拥优抚安置工作继续加强。

文化事业和文化产业蓬勃发展。潮剧发展和改革初见成效，群众文艺进一步繁荣。潮剧《东吴郡主》等一批文艺精品获得国家、省级奖项，潮州大锣鼓《六国封相》获“中国民间文艺山花奖”。潮剧、潮州音乐、英歌舞、剪纸进入第一批“国家级非物质文化遗产”推荐项目名单。市图书馆装修工程完成。哲学社会科学、文学艺术、新闻出版和广播电视繁荣发展。文化市场不断规范，音像、印刷、文具等优势文化产业加快发展。

卫生、体育、人口计生等工作有新进步。城乡公共卫生体系不断完善，市疾病预防控制中心易址建设，非典、禽流感、艾滋病等重大传染病防控工作得到强化。农村新型合作医疗覆盖面扩大。制定建设体育强市实施意见，群众体育活动广泛开展，被授予“全国游泳之乡”称号。完成经济普查工作。完成省下达的年度人口控制计划，全年人口自然增长率5.49‰。殡葬改革完成省下达的目标任务。妇女儿童、残疾人、老龄和慈善事业加快发展。档案、保密、方志、人防、气象、地震等各项事业有新进步，统计、审计、外事、侨务、对台、民族、宗教等工作取得新成绩。———和谐汕头建设开局良好

城乡居民生活水平进一步提高。中心城区居民人均可支配收入10630元，增长7%；农村居民人均纯收入4321元，增长5.7%。城乡居民储蓄存款余额733.5亿元，增长14.3%。居民消费价格指数上涨2.5%，控制在合理上涨区间。

就业再就业和社会保障工作扎实开展。全年新增就业岗位6.2万个，城镇登记失业率为3.1%。“十万农村青年就业培训工程”启动。社保基金征缴和监督进一步加强，企业养老统筹基金实现当期收支平衡，困难企业职工社保关系接续等政策逐步完善。城乡最低生活保障和社会救助工作不断加强。

涉及人民群众切身利益的热点难点问题逐步解决。加快推进“千村扶贫”工程和农村危房改造，为农村危房户、水库移民、低保困难户和残疾人修建1592套住房。完成24宗农村饮水解困工程建设，解决农村4.3万人饮水困难。解决3500套商品房发证遗留问题。为劳动者追回被拖欠工资1253万元，清偿历史拖欠征地补偿款9665万元，努力解决建设领域政府项目拖欠工程款问题。认真贯彻实施《信访条例》，群体性事件的防范处置工作得到加强。全面推进社会治安综合治理，积极创建平安和谐社区。持续开展打击暴力性犯罪、“两抢一盗”犯罪专项斗争，坚决扫除“黄赌毒”，严厉打击“法轮功”等邪教组织。认真处置潮南区“6·10”特别重大火灾事故，狠抓整改，安全生产工作得到加强。制定黄厝围易燃易爆物品库区企业整体搬迁规划方案。整顿规范市场经济秩序工作深入推进。

绿色汕头建设初见成效。我市被列入全省建设节约型社会试点城市，贵屿镇被列入第一批国家循环经济试点单位。南澳清洁能源基地建设步伐加快。自然湿地保护、森林资源保护和林业生态建设得到加强，联合国海洋生物多样性管理项目启动，完成造林作业面积1.7万亩。韩江水质保护和其他江河整治得到加强，重点污染源全面达标工作扎实推进。———依法行政工作取得新进展

民主建设稳步推进。认真执行市人大及其常委会各项决议并向其报告工作，坚持向人民政协通报情况，自觉接受监督。人大代表议案（建议）68件、政协提案132件办理完毕。加强与工会、共青团、妇联等群众团体的联系，广泛听取社会各界的意见和建议，科学民主依法决策机制进一步完善。建立政府新闻发布制度，扎实推进政务公开。全面完成1042个村（居）委会换届选举任务，落实农村干部工资补贴政策，基层民主政治建设得到加强。

依法行政工作不断增强。提请市人大常委会审议法规草案4件，颁布政府规章4件、规范性文件20件。深入贯彻行政许可法和《全面推进依法行政实施纲要》，做好第三轮行政审批制度改革后续工作，政府职能进一步转变。深入开展保持共产党员先进性教育活动，扎实推进机关效能建设，加强行政执法监督，行政效率得到提高。普法教育和法律服务工作扎实开展，全民法律意识普遍提高。落实廉政建设责任制，严肃查处违法违纪案件，认真纠正部门和行业不正之风，切实加强领导干部离任经济责任审计，廉政建设成效明显。

各位代表！我市“十五”时期国民经济和社会发展取得的成绩，是省委、省政府和市委正确领导的结果，是各级人大及其常委会和人民政协监督支持的结果，是全市人民同心同德、奋力拼搏的结果，也是社会各方面支持和帮助的结果。在此，我代表市人民政府，向全市广大工人、农民、知识分子、干部职工、驻汕人民解放军、武警官兵、人民警察和社会各界表示崇高的敬意！向关心和支持汕头建设的港澳台同胞、海外侨胞和国际友人表示衷心的感谢！

回顾“十五”时期的发展历程，我们的主要体会是：必须狠抓发展第一要务，充分发挥自身优势，不断深化改革开放，坚定不移地走工业强市、实业兴市的发展路子，促进三次产业协调发展，努力建设区域性中心城市和经济强市；必须加强经济社会管理，弘扬诚实守信的社会风尚，大力整顿规范市场经济秩序，促进经济社会持续健康发展；必须充分调动和发挥各方面积极性，正确处理历史遗留问题，理顺内外关系，形成加快发展的强大合力；必须转变政府职能，改进机关作风，提高行政效能，努力建设廉洁勤政务实高效政府；必须坚持以人为本、执政为民，努力改善人民生活，维护社会和谐稳定，让广大人民群众得到更多实惠。

在肯定成绩的同时，我们也清醒地认识到，我市经济社会发展中仍存在不少困难和问题。从目前看：一是投资拉力不足。投资率远低于全国、全省平均水平，重点建设项目进展缓慢。二是经济发展制约因素较多。用地、用电、用工紧张，能源、原材料价格持续上涨，企业融资依然困难，国际贸易竞争日趋激烈。三是财政收支矛盾突出。收入规模不大，税收占生产总值比重偏低；政府债务重，消化历史债务压力很大。四是经济社会管理有待加强。扰乱市场经济秩序的违法犯罪活动时有发生，城市管理和社会治安管理存在薄弱环节，安全生产存在不少隐患，交通拥堵现象时有出现。从长远看：“十五”期间主要经济指标未能完成预期目标，给今后发展带来很大压力；经济持续增长的动力不足，产业结构层次较低，经济增长方式粗放；人口、资源、环境与经济发展不够协调，可持续发展任务艰巨；政府职能尚未得到根本转变，行政效能不高；城乡二元结构矛盾突出，农村居民收入增长较慢等。对这些问题，必须高度重视，认真采取措施加以解决。“十一五”时期的奋斗目标“十一五”时期，是我市进一步落实科学发展观，实现跨越式发展的重要时期。在新的发展阶段，我们既面临发展新机遇，也面临严峻挑战，总体上面临有利的发展环境和条件。和平、发展、合作已成为时代潮流，经济全球化进展迅速，国际产业重组和生产要素流动加快。我国经济持续快速发展，市场经济体制进一步完善，改革开放向纵深推进，区域合作蓬勃发展。省委、省政府把扶持东西两翼发展作为建设经济强省的一个战略举措，强化汕头作为东翼区域性中心城市的地位。我市经济稳步发展，广大干部群众谋求加快发展的愿望十分强烈。这些都有利于我市进一步加快发展。与此同时，我国加入世界贸易组织的过渡期即将结束，国际贸易保护主义加剧，国内区域竞争日趋激烈，我市一些深层次矛盾和问题尚未从根本上得到解决，增加了我们的竞争压力。我们必须认清形势，抢抓机遇，全力以赴，攻坚克难，努力推动汕头经济社会全面协调可持续发展。

根据中共十六届五中全会和省委九届七次全会精神，市委八届五次全会提出《中共汕头市委关于制定全市国民经济和社会发展第十一个五年规划的建议》，描绘了我市“十一五”时期发展的宏伟蓝图。据此，市政府组织编制了《汕头市国民经济和社会发展第十一个五年规划纲要（草案）》，提出以建设现代化港口城市、区域性中心城市和生态型海滨城市，实现全市人民富裕安康为总目标，坚持以人为本，狠抓发展第一要务，着力提高自主创新能力，着力调整优化经济结构，着力转变经济增长方式，努力打造新兴制造业基地、临港工业基地、综合服务业基地和现代效益农业基地，把经济社会发展切实转入科学发展轨道，为实现跨越式发展、全面建设小康社会打下坚实的基础。全市生产总值年均增长10%以上，人均生产总值到2010年比2000年翻一番；经济增长的质量和效益明显提高，增长方式进一步转变，单位生产总值能源消耗比“十五”期末降低13%以上。社会事业全面发展，精神文明与民主法制建设不断加强，生态环境得到保护，人民生活总体达到宽裕型小康水平。

实现“十一五”目标，必须以邓小平理论和“三个代表”重要思想为指导，以科学发展观统领经济社会发展全局，努力做到“六个坚持”：

一是坚持能快则快，快中求好，协调发展。发展是第一要务，是永恒的主题。必须强化能快则快的发展意识，卧薪尝胆，加快发展。进一步落实又快又好的发展要求，实现速度与质量、效益、结构相统一，经济发展与人口、资源、环境相协调。

二是坚持内外结合，练好内功，用好外力。要自强不息，苦练内功，进一步优化经济结构，转变经济增长方式，保持经济发展的强大内动力。要海纳百川，兼容并蓄，积极引进项目、资金、人才和先进技术，把一切愿意在汕头投资发展的经济变成汕头经济，推动经济加快发展。

三是坚持改革创新，增创发展新优势。改革创新是城市发展的不竭动力。必须用改革创新的意识加快发展，用改革创新的精神推进工作，努力推进思想观念创新、发展模式创新、体制机制创新，以创新增优势，以改革促发展。

四是坚持统筹城乡发展，建设社会主义新农村。解决“三农”问题是经济社会发展的长期任务。必须按照“生产发展、生活宽裕、乡风文明、村容整洁、管理民主”的要求，认真贯彻工业反哺农业、城市支持农村和“多予、少取、放活”的方针，走以工促农、以城带乡，城乡互动协调发展的新路子。

五是坚持环境保护和资源节约，促进经济社会可持续发展。必须大力推动全社会节能降耗，节约集约利用资源，发展循环经济，保护生态环境，努力建设资源节约型、环境友好型社会。

六是坚持关注民生，着力解决人民群众最关心、最直接、最现实的利益问题。群众利益无小事。必须始终把人民群众的安危冷暖放在心上，真正做到权为民所用，情为民所系，利为民所谋。

各位代表，展望“十一五”，我们充满信心。我们相信，在全市人民的共同努力下，一个充满生机活力的新汕头，必将屹立在南海之滨！2006年的工作安排2006年是实施“十一五”规划的开局之年。政府工作的总体要求是：高举邓小平理论和“三个代表”重要思想伟大旗帜，坚持以科学发展观统领经济社会发展全局，全面贯彻中共十六届五中全会、中央经济工作会议和省委九届七次、八次全会精神，认真落实市委八届五次、六次全会的工作部署，以结构调整为主线，以提高经济增长质量效益为核心，以促进城乡区域协调发展为重点，以提高自主创新能力为突破，深化改革，扩大开放，不断推进经济建设、政治建设、文化建设和社会建设，实现经济社会又快又好发展。2006年全市经济社会发展的主要预期目标是：生产总值增长11%，单位生产总值能耗下降3%，社会消费品零售总额增长13%，全社会固定资产投资额增长11%，外贸出口总额增长12%，居民消费价格总水平上涨3%左右，地方财政一般预算收入增长12%，城镇登记失业率控制在3.5%以下，人口自然增长率控制在7.01‰以下，城镇居民人均可支配收入增长7%，农村居民人均纯收入增长5%。

实现全年目标，必须抓好以下十个方面工作：

一、增强自主创新能力，推动经济结构调整和增长方式转变

着力提高自主创新能力。加快以企业为主体的创新体系建设，开发更多具有自主知识产权的技术和产品。加快建立中小企业科技投融资、风险投资机制，大力培育自主创新型中小企业。加强以科技园、产业基地和专业镇为主体的科技孵化基地建设，培育发展行业科技研发中心。实施知识产权战略、技术标准战略和名牌带动战略，加快发展自主品牌，扩大名牌效应。 着力调整优化工业结构。坚持走新型工业化道路，促进传统、轻型化工业向特色、适度重型化工业转变。用高新技术和先进适用技术改造提升纺织服装、工艺玩具、化工塑料、食品加工等传统优势产业，提高规模以上企业占全市企业的比重，提高高新技术产品产值在全市工业产值中的比重。以印刷包装机械、食品加工机械、塑料机械和电力设备、医疗设备为重点，推动装备制造业加快发展。推进中医药强市建设，发展中医药产业。继续推进高新区、保税区和金平民营科技园、龙湖民营科技园、澄海岭海工业区规划建设和设施配套，引导产业集聚，发展特色产业园区。

加快发展临港工业和港口经济。充分利用岸线资源和港口资源，重点发展石化、能源等临港工业，培育战略性产业。抓紧潮阳、潮南、澄海、南澳等区县港区规划和汕头港总体规划修编，做好广澳港区30万吨级原油码头、10万吨级集装箱码头的前期论证工作。加快广澳港区2个5万吨级码头建设，争取5万吨级航道开工建设。积极发展港口物流业，大力开发腹地货源。加快电网规划，加快谷饶、苏南输变电工程建设和南澳过海电缆项目前期工作。抓紧华能海门电厂一期工程立项，争取年底开工建设。积极推进LNG项目前期工作。争取60万千瓦压油改煤电厂项目尽快选址立项。洽谈跟踪聚苯乙烯、特种汽车、造纸等项目。

加快发展现代服务业。加快传统服务业向现代服务业转变，以工业制造业带动服务业，以服务业促进工业制造业发展。抓紧规划建设工艺玩具、服装内衣等一批物料、辅料配件大型专业市场。办好玩具展等特色展会，培育区域性会展品牌。规划建设美食城和珠宝商城，加快建设蓝水星主题公园等旅游项目，做好迎接中国优秀旅游城市复核工作。发展和规范专业中介服务业，提升商务服务能力。拓展金融保险业，加快发展信息服务业。发挥“建筑之乡”优势，支持建筑业开拓国内外市场。适度发展房地产业，促进房地产市场健康发展。

二、以三条经济带为重点，加快城市规划建设

做好城市规划编制。完成土地利用总体规划修编，加快近期建设规划编制。整合市域各区总体规划，加强澄海、潮阳、潮南规划编制工作。完成新溪、外砂片区分区规划。编制重点区域控制性详细规划。抓紧做好南滨路片区规划调整，启动南滨路片区开发建设。

加快三条经济带的规划和建设前期工作。积极推进东部城市经济带前期工作，抓紧河口整治工程项目可研报告编制和报批，抓紧河口整治、海堤、市政大道、新城区的规划设计，做好建设准备工作，争取在“十一五”前期实施。以工业经济带规划建设为重点，提升园区建设水平。依托广澳港区、潮阳港区，深化濠江石化园区、潮阳经济技术开发区、潮南井都陇田片区的规划组合，为引进临港工业和大型装备制造业提供良好的空间条件。按照带中区、区中园的思路，在工业经济带规划台商投资工业园区、珠三角产业转移工业园区、珠宝加工区等专业园区，通过项目带动工业经济带及园区的建设。积极开展西部生态经济带前期调研工作。

抓紧交通基础设施建设。完成深汕高速公路东段大修工程。加快金凤路桥、国道324线4座大桥改造、省道谷饶－贵屿路段和揭海公路、潮南陈沙公路建设。协力推动汕揭梅高速公路汕头段建设，抓紧做好机场路拓宽改造工程，筹建南澳大桥。积极配合做好厦深沿海铁路汕头段规划设计和筹建，开展广澳港区疏港铁路规划前期工作。抓紧金砂路东延和汕樟北路续建工程建设，实施迴澜桥改造。加快启动海滨路西段、中段和中山东路拓宽改造工程，做好金砂路拓宽和濠洲路建设前期工作。

抓紧水利、市政设施建设。抓紧实施潮阳、潮南引韩供水工程，争取南澳县引韩供水工程早日立项。加快实施潮阳金关围、澄海苏溪围海堤工程、南澳城防工程，继续推进韩江下游出海口闸改建加固，加快实施小型水库除险加固、机电排灌和水库移民遗留问题等3个省人大议案。加快旧城改造步伐，加强“城中村”的规划、改造、建设和管理。加强道路设施建设和交通管理，大力发展城市公共交通，努力改善交通拥堵现象。推进断头路、水浸街和中山公园的改造建设。抓好绿化美化净化和城市亮化工程。

三、发展效益农业现代农业，加快社会主义新农村建设

大力发展效益农业、现代农业。集中力量建设和完善一批农田水利基础设施。加快农业科技进步，引进推广新技术、新品种，提高土地利用率、土地产出率和劳动生产率。切实加强耕地保护，提高粮食生产能力。加快五大优势农业区建设，发展壮大特色农业基地。引导农民兴办专业合作经济组织，扶持农业、渔业龙头企业发展壮大。大力发展农产品加工和流通业，建立健全农产品市场销售体系。加强农业示范区建设，推进农业标准化工作。健全农产品质量安全监测体系，做好高致病性禽流感等重大动植物疫病防控工作。加快海门国家中心渔港建设，抓紧申报云澳国家中心渔港、达濠国家一级渔港建设项目。继续开展人工鱼礁建设和资源增殖放流，发展海洋渔业。

加快城镇化进程。做好镇、村两级建设规划，全面完成中心镇总体规划修编。落实扶持中心镇发展的各项政策措施，发挥中心镇在城镇化进程中的龙头带动作用，以工业化带动城镇化，以城镇化促进工业化。

加强农村基础设施建设。抓好行政村通水泥路工程建设。加快实施农村安居工程，完成年度农村危房改造任务。实施农村饮水安全工程。加大老村旧镇改造力度，完善农村道路、电网、通讯、饮水、生活污水处理等基础设施，抓好农村改路、改水、改房、改厕、改灶。

全面推进农村各项事业。推进农村开发性扶贫，综合解决农村“一保五难”问题。从今年秋季起在全市农村全面实行免费义务教育，免除所有农村户籍学生义务教育阶段杂费。加强新型农村合作医疗管理，提高筹资标准和补偿水平。创新农村医疗体制，开展农村卫生站规范化建设试点工作，逐步实现农民“小病不出村”。继续深化乡镇综合配套改革，稳妥推进县乡财政体制改革。切实加强农民负担监管，加强农村财务管理，推行村账镇管。加快完善农村土地二轮承包工作。

四、集聚内外优势，促进内外源型经济协调发展

大力发展民营经济。全面落实扶持民营经济发展的政策措施，着力解决制约民营企业发展的突出问题。构建面向中小企业的社会化服务体系和信用担保体系，支持民营企业体制创新、技术创新、管理创新。引导民间资本进入公用事业、基础设施、社会事业，鼓励民营企业参与国有企业改革。

加强区域经济协作。加强区域间产业布局、基础设施建设、资源开发等方面的协作，加强与国内友好城市的经贸合作，实现与闽粤赣十三市经济协作区、泛珠三角经济圈、海峡西岸经济带各城市优势互补，共同发展。组织企业参加各类经贸活动，积极开拓国内市场。

扩大有效招商。发挥侨乡优势、对台优势，主动承接国内外产业转移，积极引进跨国公司和大型项目，提高引资质量。做好招商引资后续服务工作，全面清理已签约项目，提高履约率。鼓励现有企业增资扩产，做大做强。培育发展社团和行业协会，推动招商活动由政府主导逐步向行业协会、商会主导转变。

转变外贸增长方式。巩固传统外贸市场，大力拓展俄罗斯、中东、东欧、非洲、南美等新兴市场。鼓励和扶持拥有自主知识产权、自主品牌的商品和高新技术产品出口。扩大进口规模，积极做好我市急需的能源、重大设备和重要原材料进口。积极推进电子口岸建设，优化通关环境，提高通关效能。建立健全应对国际贸易壁垒和贸易摩擦的工作机制。

五、坚持改革创新，增创加快发展的体制机制优势

加快国企改革步伐。以产权制度改革为核心，以职工安置为重点，分类指导、分类推进国有企业改革。向社会公布一批改革企业目录，鼓励非公有资本参与企业改制、重组。完善政策，集中力量，加大投入，着力推进一批优质企业和急需改革企业实施改制，力争市属国企改革取得较大突破。妥善处理企业改革中的矛盾和问题，维护社会稳定。

深化投融资体制改革。全面实施企业投资项目核准制和备案制。加快推进城市资源市场化、资本化运营，探索垄断行业和公用事业改革，选择一批市政设施和各类特许经营权进行招投标和公开拍卖。积极推进市区管道燃气特许经营权市场化运作。完善路桥收费改革。完成上市公司股权分置改革。加大上市公司培育力度，推动企业上市直接融资。

强化市场在公共资源配置中的基础性作用。清理、盘活闲置土地，拍卖、统征、储备一批土地。竞争性工业土地使用权逐步实行招标拍卖挂牌公开交易，经营性土地使用权全面实行市场公开交易，逐步建立起统一、开放、规范、有序的土地市场体系。推行国有、集体所有房产入市公开挂牌、拍卖交易制度。规范户外广告、公共停车场、市区咪表停车管理。

六、建设资源节约型城市，促进经济可持续发展

大力节约能源资源。强化资源节约意识，推进节约型企业、社区、学校、机关建设。加强能源资源综合利用，促进重点耗能行业和企业节能降耗，促进高效节能产品的研发和推广。加强韩江等水源保护，大力节约用水，建立统一协调的水资源管理体系，提高水资源利用水平。调整土地结构和用地布局，科学配置土地资源，节约集约利用土地。

积极发展循环经济。大力推进贵屿循环经济试点工作，按照政府组织协调、企业实施、市场化运作的原则，高标准、高起点规划建设产业示范园区。加快风能发电等绿色能源项目建设，把南澳建成清洁能源基地。

加强环境保护和生态建设。重点加快6家污水处理厂和配套管网工程以及6座垃圾填埋场建设，争取通过国家环保模范城市整改复查。做好垃圾焚烧处理厂BOT运作的前期工作。加强生态林业和自然保护区建设，加强风景名胜区保护管理。保护海洋资源，依法加强海域、岸线保护性开发和汕头湾生态环境保护。开展创建全国环境优美乡镇、省级生态示范区和市级生态文明村活动，积极创建“国家园林城市”、“国家生态城市”。

七、加强财税金融管理，为加快发展提供财政金融支持

完善公共财政体制。优化财政支出结构，加大对农业、教育、科技、文化、公共卫生、社会保障等的投入。全面实施部门预算和收支两条线管理，完善国库集中收付制度，扩大政府采购规模和范围。加强财政监督和审计监督，保证财政资金稳定、安全、高效运行。健全政府投资决策机制，依法规范政府举债行为，合理控制债务规模，逐步消化政府债务。抓好机关事业单位资金清理收缴和资产、土地的清理处置工作。

加强税收征管。坚持依法治税，实行科学化、精细化管理，建立健全税收管理员制度，积极探索税收执法检查新模式，确保应收尽收。积极涵养税源，维护税负公平，优化纳税服务。强化各级各部门协税护税意识，严禁越权干预税收。加大税法宣传，营造依法诚信纳税的良好氛围。

加强和改善金融服务。积极争取政策性银行贷款、商业银行贷款、国债资金和专项资金支持。进一步推动银企合作，引导金融机构加大对地方重点建设项目和基础行业的信贷支持。严厉打击各种逃废金融债务行为，营造诚实守信的金融生态环境。深化农村信用社改革，积极稳妥推进汕头市商业银行等停业整顿金融机构最终处置工作。

八、统筹社会事业协调发展，全面提升公共服务功能

切实加强精神文明建设。深入开展“爱国、守法、诚信、知礼”现代公民教育，加强青少年思想道德建设。弘扬和实践“海纳百川、自强不息”的汕头精神，增强城市凝聚力，塑造城市新形象。增强家园意识和公共卫生意识，推进文明社区、文明村镇、文明行业建设，开展全民读书月活动，加强科普工作，倡导科学、健康、文明生活方式。

着力抓好教育和人才工作。优先发展教育，进一步巩固提高“普九”水平。加快校舍建设步伐，推进学校布局结构调整。加强师资队伍建设，加快高中发展步伐，扩大办学规模，增加优质学位。抓紧汕头一中新校区建设，确保今年秋季招生。积极发展职业教育和成人教育，鼓励和规范发展民办教育。支持办好汕头大学，认真办好汕头职业技术学院。创新引才用才机制，加大人才柔性引进力度，不断拓展引才用才空间，优化人才发展环境。

大力发展文化事业和文化产业。弘扬优秀传统文化，大力推进潮剧发展和改革。加强潮汕历史文化研究，做好潮剧发展促进会和潮剧发展专项基金的筹备工作。加强文化基层基础工作，开展丰富多彩的群众文化活动。抓紧完善市图书馆新馆配套设施，确保今年开馆。繁荣发展哲学社会科学、文学艺术、新闻出版和广播电视事业。积极稳妥推进文化体制改革，完善文化产业政策，促进文化产业快速发展。

做好人口、卫生、体育等各项社会事业。突出抓好纯二女户结扎和计生优质服务，依法征收社会抚养费，兑现计生奖励政策，力争全市人口计生工作达到全省中等水平。大力推进殡葬改革，完成火化率100%目标。推进医疗体制改革，规范医疗服务收费和药品购销活动。健全突发公共卫生事件应急处理机制、疾病预防控制体系和卫生监督体系，切实抓好重大传染病的防治工作，确保市疾病预防控制中心建成投入使用。执行新的国家卫生城市标准，做好迎接复审准备工作。认真办好马拉松游泳世界杯赛，积极做好第十二届省运会参赛工作。广泛开展全民健身活动，大力加强农村体育、社区体育设施建设。发展妇女儿童事业、老龄事业和慈善事业。认真落实优抚安置政策，巩固发展双拥工作成果，进一步加强国防教育、国防动员和民兵预备役工作。重视和做好民族、宗教、统计、物价、人防、侨务、对台、气象、地震、档案、保密、方志等工作。

九、关注民生，努力建设和谐社会

努力扩大就业再就业。实施积极就业政策，新增就业岗位6万个。加大宣传力度，扩大就业再就业政策覆盖面。拓宽就业渠道，健全公共就业服务体系。积极开展就业再就业培训、农村劳动力转移培训和创业培训，促进城乡统筹就业。

加快完善社会保障体系。扩大社会保险覆盖面，逐步将失地农民和灵活就业人员纳入社会保险范围，提高城乡居民社会保障水平。完善医疗保险制度。推进企业退休人员社会化管理。拓宽社保基金筹措渠道，加强运行管理，增强保障能力。实施城乡医疗救助，完善社会救助制度，实现低保对象应保尽保。

切实解决好群众关注的热点难点问题。全面落实“十项民心工程”，切实解决部分群众住房难、饮水难、入学难、打官司难、看病难等问题，不断改善群众生产生活条件。妥善解决“五保户”和特困户的生活困难。动工建设特殊教育学校，做好残疾人就业工作，推行残疾人就业保证金由地税部门征收，推动残疾人事业发展。继续开展清理拖欠征地补偿款、工程款和农民工工资等工作。按照“二级政府，三级管理，四级网络”的管理模式，落实城市管理责任制。下大力气整治脏乱差现象，努力改善市容市貌。开展食品药品专项整治，认真实施食品、药品放心工程。

全力维护社会治安稳定。积极排查调处人民内部矛盾纠纷，进一步加强和改进群体性事件的预防处置工作。严厉打击重大恶性犯罪和“两抢一盗”等多发性犯罪，解决突出治安问题。全面推进社会治安防控体系建设，加强出租屋和流动人口管理，积极创建平安和谐社区，构建社会治安长效机制。严厉打击制假贩假、走私贩私、制贩假币等各种经济犯罪，坚决打击和取缔“六合彩”赌博活动。开展保护知识产权专项行动，打击各种商业欺诈行为，加强市场价格监管，规范市场经济秩序。

狠抓安全生产。加强城市公共安全、防灾减灾等体系建设。严格落实防范重大安全事故责任制，加大安全生产执法检查和消防安全专项整治力度，加强安全生产基础设施建设，加强消防安全宣传教育，逐步建立安全生产长效机制，坚决遏制重特大安全生产事故发生。健全海上安全生产救助机制。

十、依法行政，建设廉洁勤政务实高效政府

完善民主政治制度。坚持政府向人大及其常委会报告工作、向人民政协通报情况的制度，自觉接受人大及其常委会的法律监督和人民政协的民主监督。认真办好人大代表议案、建议和政协委员提案、建议案。主动听取各民主党派、工商联、无党派人士和各人民团体的意见建议。建立健全重大事项社会公示和听证制度，完善重大决策的规则和程序，健全科学民主依法决策机制。推进电子政务建设，完善政府新闻发布制度，提高政府工作透明度。实行村务、厂务公开，加快推进村（居）民自治和企事业单位民主管理。

全面推进依法行政。转变政府职能，深化行政管理体制和行政审批制度改革，清理行政执法、公共服务事项和办事制度，进一步提高行政效率和服务水平。加强对经济运行的监测分析，完善经济风险规避防范机制和应急预警机制。改进政府管理经济的方式方法，提高政府领导经济工作的能力。建立健全突发事件应急机制，提高政府保障公共安全和处置突发事件的能力。依法管理和规范社会事务，建立健全社会利益协调机制，提高依法处置和化解矛盾的能力。提高政府立法质量，强化行政执法监督，规范行政执法行为，做好行政复议和行政应诉工作。启动“五五”普法工作。

加强勤政廉政建设。贯彻实施《公务员法》，加强和改善公务员管理。加强机关效能建设，改进机关作风。切实精简会议、文件，狠抓各项决策和工作部署的检查落实。积极推进预防和惩治腐败体系建设，落实廉政责任制和廉洁自律各项规定。强化监察和审计工作，加大违纪案件查处力度，坚决纠正损害群众利益的不正之风。

各位代表！今年是“十一五”规划的第一年，开好头、起好步至关重要。新的目标令人鼓舞，新的形势催人奋进。让我们高举邓小平理论和“三个代表”重要思想伟大旗帜，全面落实科学发展观，振奋精神，锐意创新，为把汕头建设成为经济强市和区域性中心城市而努力奋斗！
